# Molecular imaging of nestin in neuroinflammatory conditions reveals marked signal induction in activated microglia

**DOI:** 10.1186/s12974-017-0816-7

**Published:** 2017-03-03

**Authors:** Senthil Krishnasamy, Yuan-Cheng Weng, Sai Sampath Thammisetty, Daniel Phaneuf, Melanie Lalancette-Hebert, Jasna Kriz

**Affiliations:** 1 Department of Psychiatry and Neuroscience, Faculty of Medicine, Laval University, Quebec, Canada; 2 Research Centre of Institut universitaire en santé mentale de Québec, 2601 Chemin de la Canardière, Quebec, Québec G1J 2G3 Canada

**Keywords:** In vivo imaging, Inflammation, Stroke, Immune biomarkers, Nestin, Microglia

## Abstract

**Background:**

Nestin is a known marker of neuronal progenitor cells in the adult brain. Following neuro- and gliogenesis, nestin is replaced by cell type-specific intermediate filaments, e.g., neurofilaments for panneuronal expression and glial fibrillary acidic protein as a specific marker of mature astrocytes. While previous work have been mostly focused on the neuronal fate of nestin-positive progenitors, in the present study, we sought to investigate in *real time* how nestin signals and cellular expression patterns are controlled in the context of neuroinflammatory challenge and ischemic brain injury.

**Methods:**

To visualize effects of neuroinflammation on neurogenesis/gliogenesis, we created a transgenic model bearing the dual reporter system luciferase and GFP under transcriptional control of the murine nestin promoter. In this model, transcriptional activation of nestin was visualized from the brains of living animals using biophotonic/bioluminescence molecular imaging and a high resolution charged coupled device camera. Nestin induction profiles in vivo and in tissue sections were analyzed in two different experimental paradigms: middle cerebral artery occlusion and lipopolysaccharide-induced innate immune stimuli.

**Results:**

We report here a context- and injury-dependent induction and cellular expression profile of nestin. While in the baseline conditions the nestin signal and/or GFP expression was restricted to neuronal progenitors, the cellular expression patterns of nestin following innate immune challenge and after stroke markedly differed shifting the cellular expression patterns towards activated microglia/macrophages and astrocytes.

**Conclusions:**

Our results suggest that nestin may serve as a context-dependent biomarker of inflammatory response in glial cells including activated microglia/macrophages.

**Electronic supplementary material:**

The online version of this article (doi:10.1186/s12974-017-0816-7) contains supplementary material, which is available to authorized users.

## Background

Nestin is a class IV intermediate filament [1] highly expressed in the multipotent stem cells of the developing central nervous system (CNS). In the adult brain, and in physiological conditions, nestin is predominately expressed in stem cells in the subventricular zone (SVZ) or at low levels in the choroid plexus, and it is considered as a marker of progenitor cells. To date, it has been widely established that nestin-positive progenitor cells may differentiate into a variety of CNS cells including neurons, astrocyte, and oligodendrocytes [2, 3]. Following neuro- and gliogenesis, nestin is replaced by the cell type-specific intermediate filaments, e.g., neurofilaments for panneuronal expression and glial fibrillary acidic protein (GFAP), a marker of mature astrocytes. Surprisingly, recent evidence revealed that in the adult mouse brain, following resident microglia depletion/repopulation experiments, the majority of the newly differentiated Iba-1-positive cells were also expressing nestin [4]. This suggests that a subpopulation of nestin-positive cells may potentially differentiate into fully ramified resident microglia [4]. Microglia are the principal immune cells in the brain and previous evidence strongly suggests that neuroinflammation and innate immune signals may modulate neurogenesis, CNS progenitor proliferation, migration, differentiation, survival, and incorporation of newly born neurons into the CNS circuitry [2, 5]. Importantly and in accordance with Rolls and colleagues [6], by using in vivo bioluminescence/biophotonic imaging of innate immune response in the Toll-like receptor 2 (TLR2) reporter mouse model generated in our laboratory, we observed that immune signals in certain conditions could be detected not only in microglia but also in doublecortin (DCX)-positive neural progenitors [7].

While previous work have been mostly focused on the neuronal fate of nestin-positive progenitors, in the present study, we sought to investigate in *real time* how nestin signals and its cellular expression patterns are controlled in the context of neuroinflammatory, innate immune challenge and ischemic brain injury. To visualize the nestin signal from the brains of living mice, we created a transgenic model co-expressing reporter genes luciferase (luc) and green fluorescent protein (GFP) under transcriptional control of the murine nestin gene promoter. The advantage of the dual reporter system emerged from the fact that fluorescence signals can be used to achieve microscopic resolution and detection of the GFP signals from the specific cell subtypes, while bioluminescence, owing to favorable emission spectra of luciferase (above 620 nm), is optimized for live whole animal imaging [7, 8]. In the present study, we describe a novel transgenic model system for in vivo bioluminescence and fluorescence imaging of nestin and we report here a context- and injury-dependent induction and a marked shift in cellular expression patterns of nestin. While in physiological conditions the nestin signal (and/or GFP expression) is indeed restricted to neural progenitors (NPGs) in their typical niche regions, the induction and/or cellular expression patterns of nestin markedly differ in neuroinflammatory conditions. Following innate immune challenge by lipopolysaccharide (LPS) injection and in response to ischemic injury, we observed a marked shift in the nestin cellular expression patterns towards activated microglia/macrophages and astrocytes. Based on our findings, we propose that nestin may have a role as a context-dependent biomarker.

## Methods

### Generation of transgenic mice

The rat promoter of the nestin gene was amplified from the recombinant plasmid pNERV [9] using a high-fidelity polymerase. The 5.1-kb fragment was subcloned into a TOPO vector, sequenced, and inserted into the recombinant plasmid pIRES-Luc2-AcGFP. To preferentially direct the transgene expression into CNS, a 654-bp fragment containing the second intron of the rat nestin gene was also amplified from the pNERV recombinant plasmid and inserted between the Cla1 restriction sites at the end of the final construct [1]. The construct pIRES-rNestin-promoter-Luc2-AcGFP-int2 was removed from its host plasmid, purified, and used for microinjection into the pronuclei of fertilized single cell C57BL/6 albino mouse embryos. The integrity of the final construct was verified by sequencing. Transgenic mice were generated in the Transgenic and Knockout Facility of the Research Center of the Centre Hospitalier de l’Université Laval (CHUL). Transgenic animals were genotyped by polymerase chain reaction (PCR) detection of the luciferase reporter gene as previously described (primers 5′-GGCGCAGTAGGCAAGGTGGT-3′ and 5′-CAGCAGGATGCTCTCCAGTTC-3′) [7, 8].

### Surgical procedure

#### Experimental ischemia

Unilateral transient focal cerebral ischemia was induced as previously described [7, 10, 11] by intraluminal filament occlusion of the left middle cerebral artery (MCA) for 90 min followed by a 2-week reperfusion period. The surgery was carried out on adult, 2–3-month-old (20-30 g) transgenic nestin-luc-GFP male mice. The animals were anesthetized with 2% isoflurane in 100% oxygen at a flow rate of 2 L/min. To avoid cooling, the body temperature was regularly checked and maintained at 37 °C with a heating pad. The correct placement of the filament was confirmed by Laser Doppler measurements (PF5001, Perimed, Sweden) [7, 10, 11]. As previously described, to additionally confirm successful MCA occlusion (MCAO), at 6 and 24 h after surgery, the animals were examined for early neurological deficits [11, 12]. The body temperature was maintained at 37 °C with a heating pad for 1 week after ischemia. To assess induction of the nestin bioluminescence/biophotonic signals, the animals were imaged over the 2-week period following MCAO.

#### In vivo bioluminescence imaging

As previously described [7, 13], the images were gathered using the IVIS 200 Imaging System (PerkinElmer, Hopkinton, Massachusetts). The luciferase substrate D-luciferin (150 mg/kg in 0.9% saline) was injected intraperitoneally (i.p.) 20 min prior to the imaging session. The mice were anesthetized with 2% isoflurane in 100% oxygen at a flow rate of 2 L/min, placed in the heated light tight imaging chamber, and maintained anesthetized by constant delivery of the 2% isoflurane–oxygen mixture at 1 L/min through an IVIS anesthesia manifold. To obtain baseline expression measurements, all animals were imaged before and then 24 h, 72 h, and 5, 7, 10, and 14 days following the injury. The light output was quantified by determining the total number of photons emitted per second (p/s) using the Living Image 4.0 acquisition and imaging software (PerkinElmer, Hopkinton, Massachusetts). Region of interest measurements on the images were used to convert surface radiance (p/s/cm^2^/sr) to source flux or total flux of photons expressed in photons per seconds. The data were represented as pseudo color images indicating light intensity (red and yellow, 5 most intense), which were superimposed over gray scale reference photographs. For the acquisition of three-dimensional (3D) images, we acquired gray scale photographs and structured light images followed by a series of bioluminescent images using different wavelengths (560–660 nm). 3D images were created using diffuse light imaging tomographic (DLIT) algorithms to reconstruct for the position, geometry, and strength of the internal light sources. The modifiable parameters were analyzed across the wavelengths, source spectrum, and tissue properties (Living Image 4.0 3D analysis software). As previously described, during all imaging sessions, the same parameters have been used across the groups and time points [13].

#### Acute and chronic inflammation model (LPS-induced inflammation)

LPS, a gram-negative bacterial cell surface proteoglycan, known also as bacterial endotoxin, has been widely used to activate the innate immune response in both the periphery and the brain. To induce acute and chronic immune challenge, a first group of mice received a single intraperitoneal (i.p.) injection of LPS (5 mg/kg body weight) (Sigma Aldrich, USA) while a second group received repetitive LPS injections (5 mg/kg body weight, every 3 days) for 2 weeks. The nestin signal was recorded by live imaging over the 2-week period (*n* = 6/group) [7]. All experimental procedures on animals were approved by the Laval University Animal Care committee and are in accordance with *The Guide to the Care and Use of Experimental Animals of the Canadian Council on Animal Care*.

#### Tissue collection and immunofluorescence

**Fig. 1:**
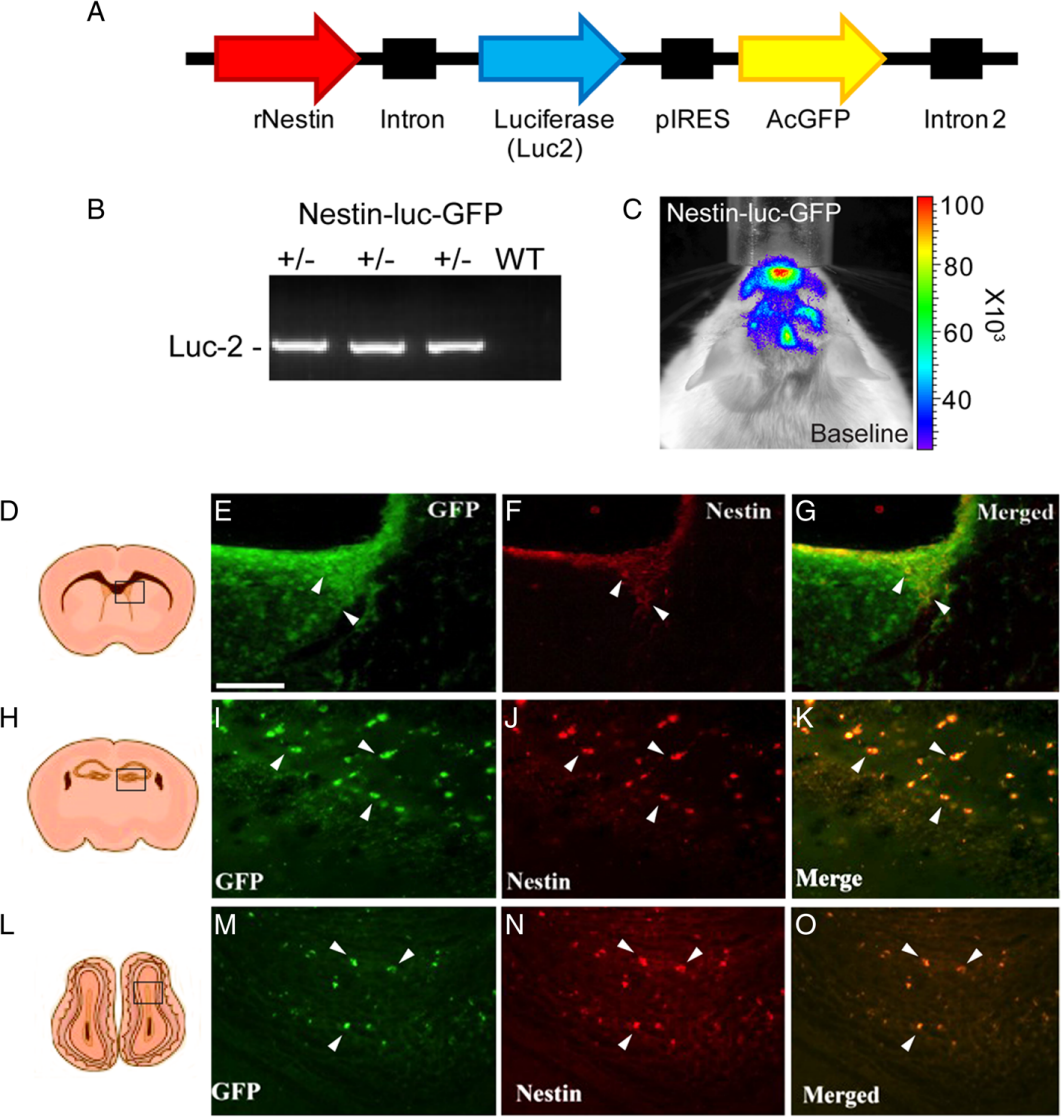
Generation and characterization of nestin-luc/GFP mice. **a** The rat promoter of the nestin gene was amplified from the recombinant plasmid. The 5.1-kb fragment was subcloned into a TOPO vector, sequenced, and inserted into the recombinant plasmid pIRES-Luc2-AcGFP. A 654-bp second intron of the rat nestin gene was also amplified from the pNERV recombinant plasmid and inserted between the Cla1 restriction sites at the end of the final construct. The construct pIRES-rNestin-promoter-Luc2-AcGFP-int2 was removed from its host plasmid and microinjected into the pronuclei of fertilized single**-**cell mouse embryos. **b** Genotyping PCR analysis for luciferase (Luc2) in the ear tissue of nestin-luc-GFP transgenic mice. **c** In vivo imaging of the photon emission in the brain of the nestin-luc-GFP transgenic mice. Immunofluorescent labeling shows the co-localization between GFP (*green*) and nestin (*red*) in nestin-Luc-GFP mice in different neurogenic regions of the brain such as the subventricular zone (**d**–**g**), dentate gyrus (**h**–**k**), and olfactory bulb (**l**–**o**) (*n* = 3 mice). The *white arrowheads* show the double positive cells. *Scale bar*: 100 μm

Animals were anesthetized by a ketamine/xylazine intraperitoneal injection (100–10 mg/kg) and then transcardially perfused with 30 mL of 0.9% saline, followed by 4% paraformaldehyde (PFA) at pH 7.4 dissolved in phosphate-buffered saline (PBS). Tissue samples were then post-fixed overnight in 4% PFA and equilibrated in PBS/30% sucrose for 48 h. Tissues were cut with a microtome (35 μm thick) and stored at −20 °C in antifreeze solution (5.75 mM sodium phosphate monobasic, 19.25 mM sodium phosphate dibasic, 30% ethylene glycol, 20% glycerol). Every sixth brain section was used for the experiments. For each animal, six sections were stained. Tissue sections from antifreeze solution were washed in PBS1X, mounted on microscopic glass slides, and then dried for 2 h under vacuum. Sections were blocked in 1% bovine serum albumin/PBS 1× and incubated overnight at room temperature using primary antibodies, mouse monoclonal nestin 1:250 (Millipore, Chemicon), 1:750 rabbit polyclonal anti-Iba-1 (Wako), 1:250 goat polyclonal anti-GFP for Fig. 3e (Santa Cruz Biotechnology), or 1:250 rabbit polyclonal anti-GFP for Fig. 1e, i, m; Fig. 3b, h, k; and Additional file 1: Figure S1D, F, and H (Millipore Chemicon), 1:500 rabbit polyclonal anti-glial fibrillary acidic protein (GFAP; Dako Cytomation) and 1:500 Doublecortin (Santa Cruz Biotechnology). After PBS 1× washes, sections were incubated in corresponding fluorescent secondary antibody (Alexa Fluor 488, 555, 595, 647) (Thermo Fisher), rewashed, and covered with Fluoromount G medium (Electron Microscopy Sciences). Images were acquired with a DVC-2000C digital camera (Thorlabs Scientific Imaging, Austin, TX) and analyzed with Stereo Investigator software (MBF Bioscience, Williston, VT). The same luminosity and exposure time were used to capture images of all the experimental groups to standardize the analysis of the entire experiment. The cell counts were performed using Neurolucida software (magnification 20×) following the protocol described by Ulrich-Lai et al. [14] that was adapted for our experiments (SVZ and DG).

#### Primary cell cultures

Primary cell cultures were obtained from the brains of PN6-PN8 wild-type pups. Brains were collected and placed in ice-cold PBS. Following mechanical dissociation, five or six brains were incubated in a 0.25% trypsin-EDTA solution (Sigma) containing 250K U/mL DNase I (Sigma). After centrifugation, the cell pellets were placed in T-75 cm^2^ flasks for 7 days at 37 °C, 5% CO_2_, in DMEM high-glucose media with 10% fetal bovine serum and antibiotic solution (Sigma). At confluence, the cells were plated onto cover slips in 24 well plates at a concentration of 40,000 cells/well in media. Cells were incubated with G-CSF 24 h later to allow adhesion. At day 4 in vitro, cells were treated with LPS (1 μg/mL) for 24 h and then fixed with PFA for immunofluorescence experiments [15].

#### Statistical analysis

Unpaired *t* test was used to compare the total photon emission in the two different ROIs in the same mouse at a same time point when the variances were equivalent between the two groups. All results are presented as mean values ± standard error of the mean (SEM). One-way ANOVA followed by Tukey’s multiple comparison tests was used for all cell counts (nestin^+^, GFAP^+^, and Iba-1^+^ cells) after immunofluorescence experiments in basal conditions, before/after MCAO, and after LPS injection. Statistical analyses were performed using the GraphPad Prism 6 software (GraphPad, La Jolla, CA).

## Results

### Generation of transgenic model for live imaging of the nestin biophotonic/bioluminescence signals

To visualize the spatial and temporal dynamics of NPG cellular responses/nestin signal in real time and from the brains of live animals, we generated transgenic mice bearing a dual bicistronic reporter system [firefly luciferase (luc) and GFP] under the transcriptional control of the nestin gene promoter. The 5.1-kb fragment of the nestin gene promoter was amplified from the recombinant plasmid pNERV and cloned into a TOPO vector then sequenced and inserted into the recombinant plasmid pIRES-Luc2-AcGFP (Fig. 1a). The feasibility of this approach was clearly demonstrated in our previous studies [7, 8]. Namely, the advantage of the dual reporter system emerged from the fact that fluorescence signals can be used to achieve microscopic resolution while bioluminescence, due to favorable emission spectra of luciferase, is optimized for in vivo imaging. Furthermore, previous studies demonstrated that the expression of nestin in the neuroepithelial cells is dependent on the presence of the transcriptional enhancer present in the second intron of the gene; therefore, to preferentially direct the transgene expression into CNS stem cells, a 654-bp fragment containing the second intron of the rat nestin gene was also amplified from and inserted between the Cla1 restriction sites at the end of the final construct [1]. Nine transgenic founders were obtained using this construct, and following validation analysis, we focused on the transgenic reporter line with the strongest signal induction and appropriate nestin-driven transgene expression. The mice developed normally and, as expected, did not develop any overt phenotype. As presented in Fig. 1b, the transgenic mice were genotyped using PCR with primers aiming at the segment of the *luc* transgene (Fig. 1b). To determine the functionality of the transgene, the animals were screened for in vivo bioluminescence signal in control, baseline conditions, following focal brain ischemia and in the context of acute and chronic neuroinflammatory conditions. We first assessed the distribution of the nestin signal in the baseline, control conditions in adult mice. As shown in Fig. 1c, in physiological conditions, the nestin bioluminescence signal was restricted to the olfactory bulb region and areas reflecting subventricular zones and hippocampus. To further validate our transgenic model, we next investigated the expression patterns of the nestin-driven transgenes luciferase and GFP. The analysis of the nestin signal by in vivo bioluminescence was followed by double-immunofluorescence analysis confirming the nestin/GFP co-localization. Moreover, the appropriate negative control experiments have been performed to additionally validate our transgenic model and in vivo imaging approaches (Additional file 1: Figure S1A–C) as well as the used immunofluorescence protocols (Additional file 1: Figure S1D–G). As shown in Additional file 1: Figure S1A–C, the in vivo imaging negative controls in wild-type (WT) mice were performed in baseline conditions and 24 h after MCAO and LPS injection. Importantly, in any of the tested conditions, we did not observe a non-specific bioluminescence signal. In addition, we also analyzed brain sections of WT mice for non-specific immunofluorescence signals in control conditions as well as following MCAO and LPS challenge. Importantly, as shown in Additional file 1: Figure S1D–G, in the tested conditions, we did not observe a non-specific immunofluorescence staining, thus further confirming the validity of our transgenic model system.

In the adult brain, neurogenesis persists in two brain regions referred to as neurogenic niches, the subgranular zone of the dentate gyrus (DG) in the hippocampus and the subependyma of the lateral ventricles called the subventricular zone (SVZ). While SVZ progenitors give rise to rostral migratory stream and incorporate into the olfactory bulb as new olfactory neurons [16], the subgranular zone progenitors migrate to the granule cell layer of the dentate gyrus and differentiate primarily into granule cells and/or interneurons [16, 17]. Importantly, the glial and neuronal progenitors are marked by expression of the intermediate filament nestin. Previous studies have demonstrated that in normal condition, there is a low level of NPG proliferation and nestin expression [16, 17]. In keeping with previous findings, analysis of the adult brains in normal conditions revealed a low basal level of the endogenous nestin protein presented in the ventricular zone (Fig. 1e–g), dentate gyrus (Fig. 1i–k), and olfactory bulb (Fig. 1m–o) and low levels of the nestin-driven GFP transgene immunoreactivity. As further revealed by double-immunofluorescence analysis, the majority of nestin-positive cells co-localize with the GFP, thus suggesting an adequate transgene expression pattern (Fig. 1e–o). Hence, in the adult mouse brain and in physiological conditions, the nestin signal expression is indeed restricted to a neurogenic niche region comprising the SVZ and DG as well as the olfactory bulb (OB).

### Cerebral ischemia is associated with strong induction of the nestin biophotonic/bioluminescence signal

**Fig. 2:**
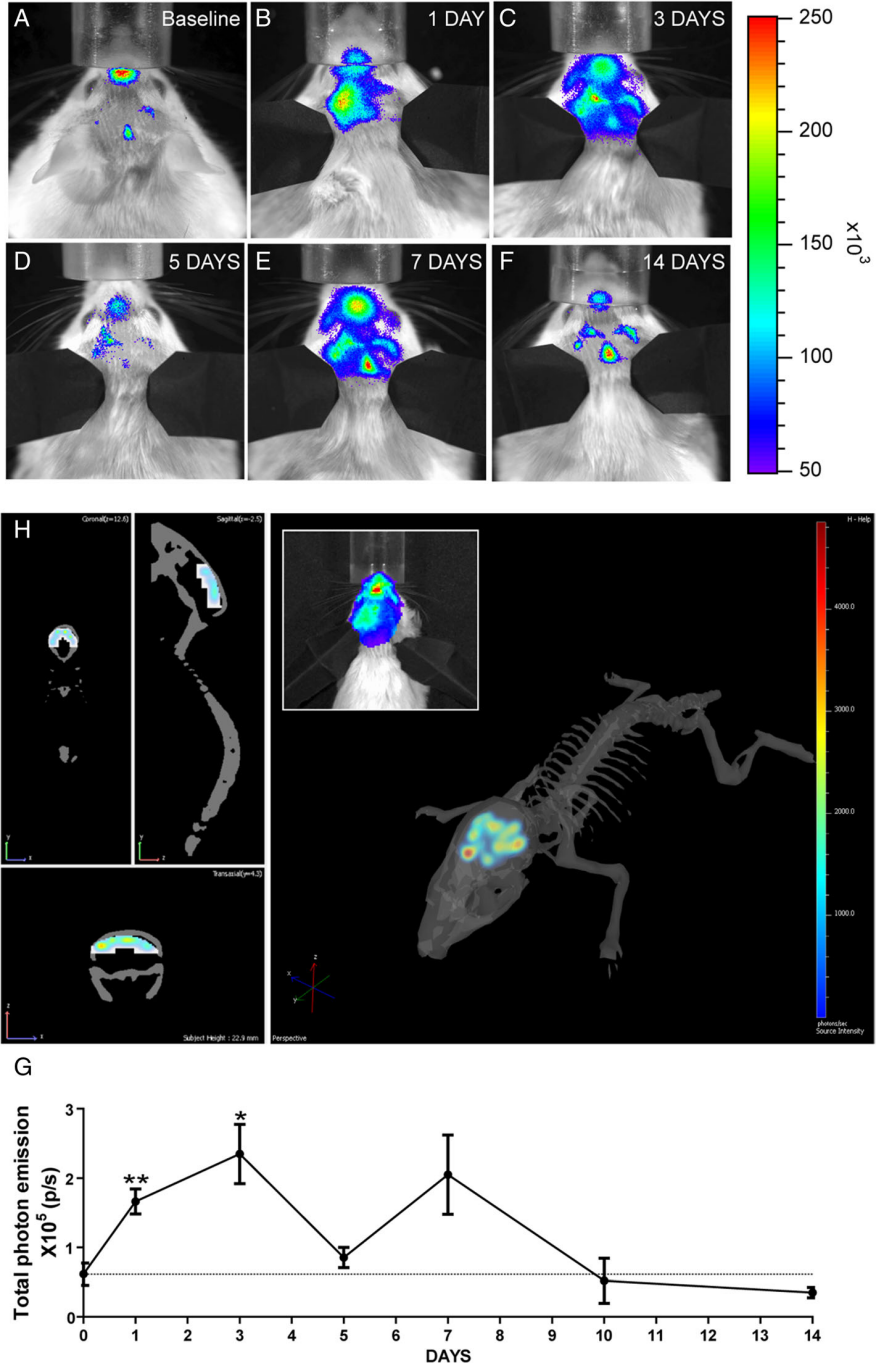
Longitudinal imaging of nestin induction/expression following transient MCAO in nestin-luc-GFP mice reveals a long-term induction of neurogenesis. **a**–**f** In vivo imaging of nestin signal induction after MCAO shows the activation of nestin promoter up to 2 weeks post-ischemia. The *color calibrations* at *right* are photon counts. Representative images showed a collection of imaging samples at six different wavelengths (560–660 nm) across the emission spectrum of the bioluminescent source (firefly luciferase) with the substantial fraction of light over 600 nm. **g** Plot of the data obtained by measuring the luciferase activity at the site of ischemic lesion (in photon per second, p/s). The *solid black line* shows the nestin induction after MCAO. A strong induction of the promoter at 24 h after MCAO (MCAO vs baseline **p = 0.0154, *n* = 5) with a peak of expression at 72 h, (MCAO vs baseline *p = 0.0347, *n* = 5) as well as smaller peak of expression at 7 days was noted. **h** 3D reconstruction of bioluminescent signal at 24 h after MCAO in nestin-luc-GFP mice. Using DLIT algorithms and structural images of the mouse, these data were transformed into 3D images. The more intense signal is seen in the frontal part of the brain of the mouse and strong activation of signal on the site of ischemia

**Fig. 3:**
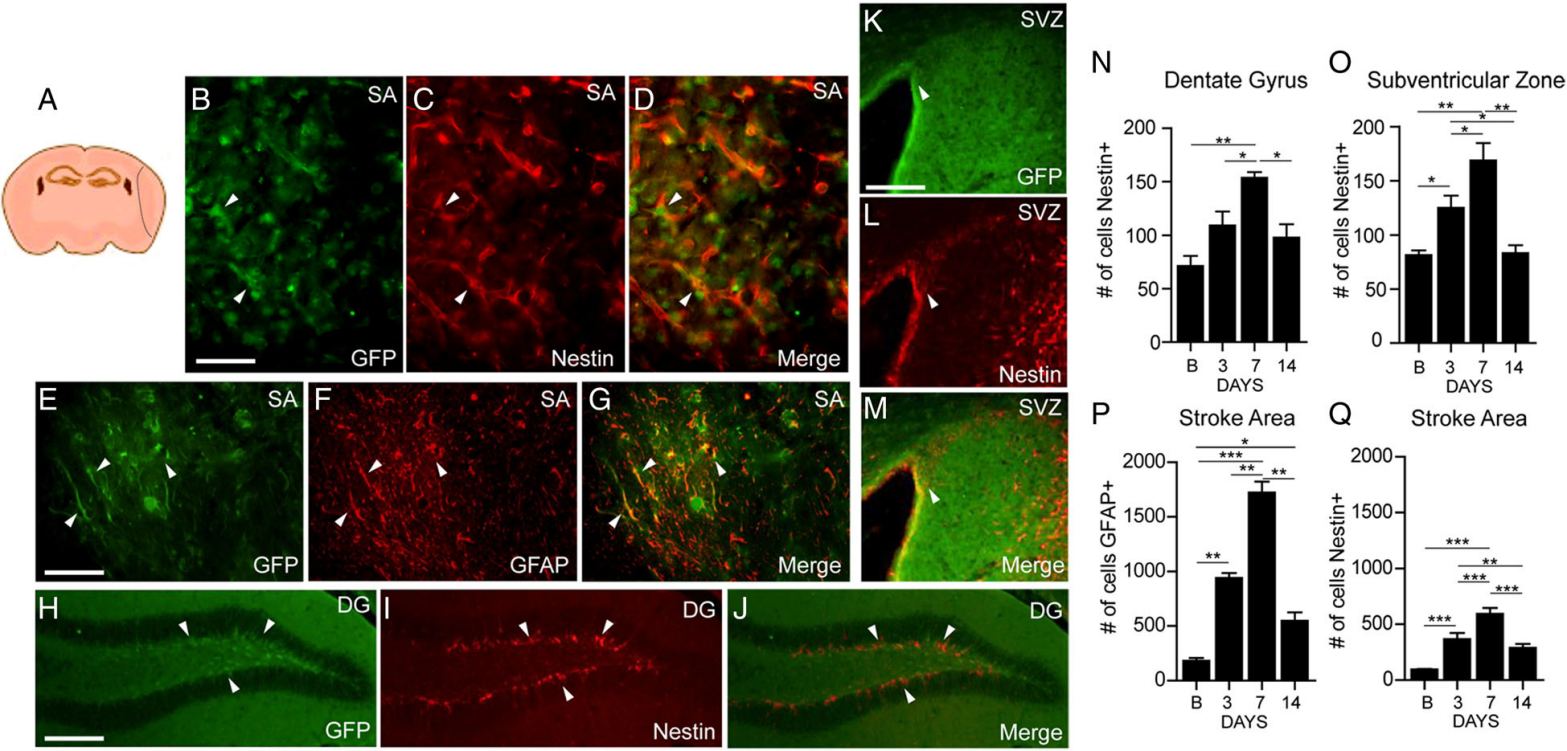
Expression of nestin-positive cells in the SVZ, DG, and stroke area (SA) regions. Schematic presentation of the stroke area (**a**). Analysis of brain sections by immunofluorescence revealed an increase in expression of nestin-positive cells of the SA (**b**–**g**), DG (**h**–**j**), and SVZ (**k**–**m**) 7 days after MCAO. Statistical analysis showed a significant increase of nestin-positive cells after MCAO, particularly at 7 days of MCAO in the DG (**n**), SVZ (**o**), and SA (**p**–**q**) (*p ≤ 0.05, **p ≤ 0.01, ***p ≤ 0.001). The number of GFAP-positive cells was also increased in the stroke area region at 3 and 7 days. The *white arrowheads* show the double positive cells. Statistical analysis was performed by one-way ANOVA followed by Tukey’s multiple test (*n* = 3–5). *Scale bar*: 100 μm

To date, it has been widely established that ischemic injury in the brain triggers adult neurogenesis in the SVZ of the lateral ventricles and the SGZ of the dentate gyrus [18–21]. Furthermore, cerebral ischemia is associated with a marked acute and chronic inflammatory response, events that may significantly affect nestin signal and/or affect its cellular expression patterns. To visualize the spatial and temporal dynamics of the nestin signal after stroke, the transgenic nestin-luc/GFP mice were subjected to 90-min transient MCAO followed by different reperfusion periods. The imaging protocol was initiated shortly before stroke (baseline level measurements), and the same animals were longitudinally imaged starting at 6 h, 24 h, and 3, 5, 7, 10, and up to 14 days after the initial ischemic injury (Fig. 2a–f). As expected, analysis of the baseline nestin signal showed low bioluminescence baseline levels. The ischemic injury induced a marked and progressive increase of the nestin signal, reaching its peak at 24–72 h and 1 week after ischemic lesion. After day 7, the intensity of the nestin signal slowly decreased reaching the baseline value at 10 days and stayed at that level till 2 weeks following stroke (Fig. 2g). As previously described, to determine whether photons detected by charge-coupled device camera after cerebral ischemia were emitted from the appropriate brain regions, we performed spectral imaging and a 3D reconstruction of the recorded signals using DLIT algorithms (see the “Methods” section) 7 days after stroke [13]. As revealed in Fig. 2h, the 3D signal reconstruction of the bioluminescent sources revealed that the nestin signal was indeed arising from the areas surrounding the brain structures normally affected by MCAO and involved in neurogenesis, including the SVZ and striatum as well as the hippocampus. These findings were further validated by immunohistochemistry analysis (Fig. 3a–j). Importantly, recorded signal distribution was consistent with our previous results obtained by in vivo imaging of different reporter mice following transient MCAO [7, 10].

**Fig. 4:**
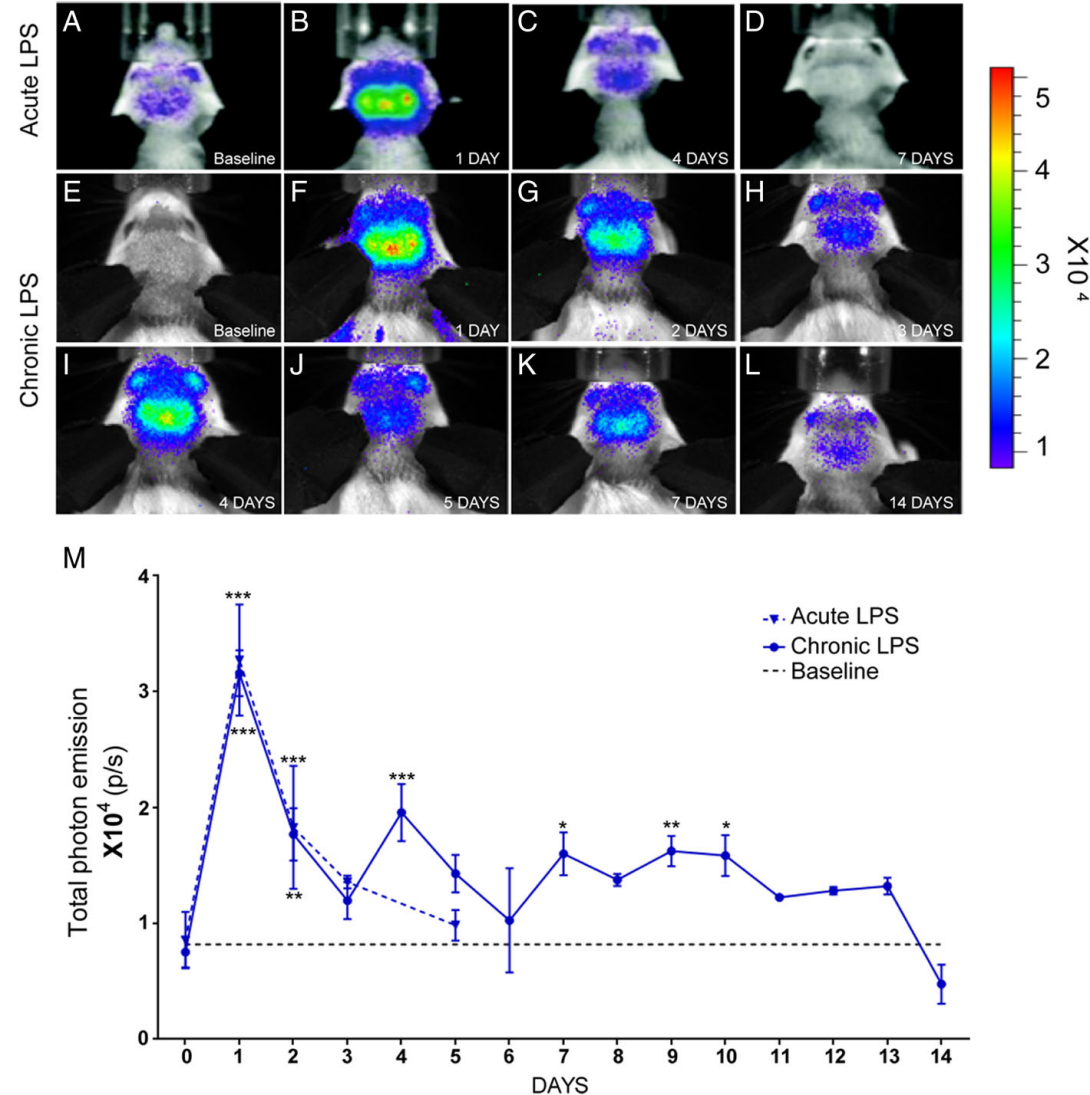
In vivo imaging of the acute and chronic inflammatory immune response in the brain of nestin-luc-GFP mice. Single LPS injection showed robust induction of the nestin signal after 24 h (**a**, **b**). The signal tends to decrease after 24 h (*dotted blue line*) (**c**, **d**). Repetitive injections of LPS (5 mg/kg) every 3 days produced a second small peak in the nestin signal intensity after 4 days (*solid blue line*) (**e**–**l**). **m** Quantitative analysis of the nestin signal by in vivo imaging after LPS injection showed a significant increase in signal intensities 24 h after challenge in both experimental paradigm (acute and chronic LPS administration protocols) (LPS vs baseline ***p ≤ 0.0001, *n* = 8–10/group). The nestin signal gradually decreased to reach baseline level at day 3. Chronic exposure of LPS resulted in fluctuation of the nestin signal expression 2 weeks after of LPS treatment (LPS vs baseline *p ≤ 0.05, **p ≤ 0.01, ***p ≤ 0.001)

Previous evidence suggests that the nestin-positive progenitors may also give rise and differentiate into GFAP-positive astrocytes; we next investigated the cellular expression/induction patterns of nestin after stroke. Based on the results obtained by in vivo imaging, i.e., peak signal induction of nestin (see Fig. 2g), the ischemic brains were collected at 3–7 days after ischemia and stained for nestin and GFP. Because previous evidence suggests a marked up-regulation of nestin in reactive astrocyte surrounding the lesion site, we first analyzed the pattern of the nestin-driven GFP expression in the peri-infarct/stroked zone. The specificity of transgene expression was then assessed by analysis of GFP expression in the cell types known to be highly responsive to brain damage. Immunofluorescence analysis of the peri-infarct region showed co-localization of the nestin-driven transgene GFP with endogenous protein or with the known astrocyte marker GFAP (Fig. 3a–g). Next, the cellular expression pattern of the GFP transgene was also assessed in the hippocampus (Fig. 4h–j) and in the SVZ (Fig. 3k–m), neuroanatomical regions known to be involved in the adult brain neurogenesis. We observed a marked up-regulation of nestin in the ipsilateral dentate gyrus at 1-week post-injury (Fig. 3n). Namely, the quantitative analysis revealed a significant increase in the numbers of the GFP and nestin-positive cells at 7 days after initial stroke. The similar pattern of the GFP and nestin immunoreactivities was observed at the SVZ region (Fig. 3o). Taken together, quantitative analysis of the GFP, GFAP, and nestin-positive cells revealed a significant increase in cell numbers starting at 3 days and peaking 7 days after stroke (Fig. 3n–q). Importantly, and as revealed by combined in vivo imaging and double-immunofluorescence data, the early peak of the nestin signal observed by in vivo imaging (biophotonic/bioluminescence imaging protocol) (24–72 h after stroke) is caused, in part, by a marked increase in the nestin signal arising from the activated astrocytes located at the peri-infarct region.

### Acute and chronic LPS treatments induce robust and transient induction of the nestin signals

**Fig. 5:**
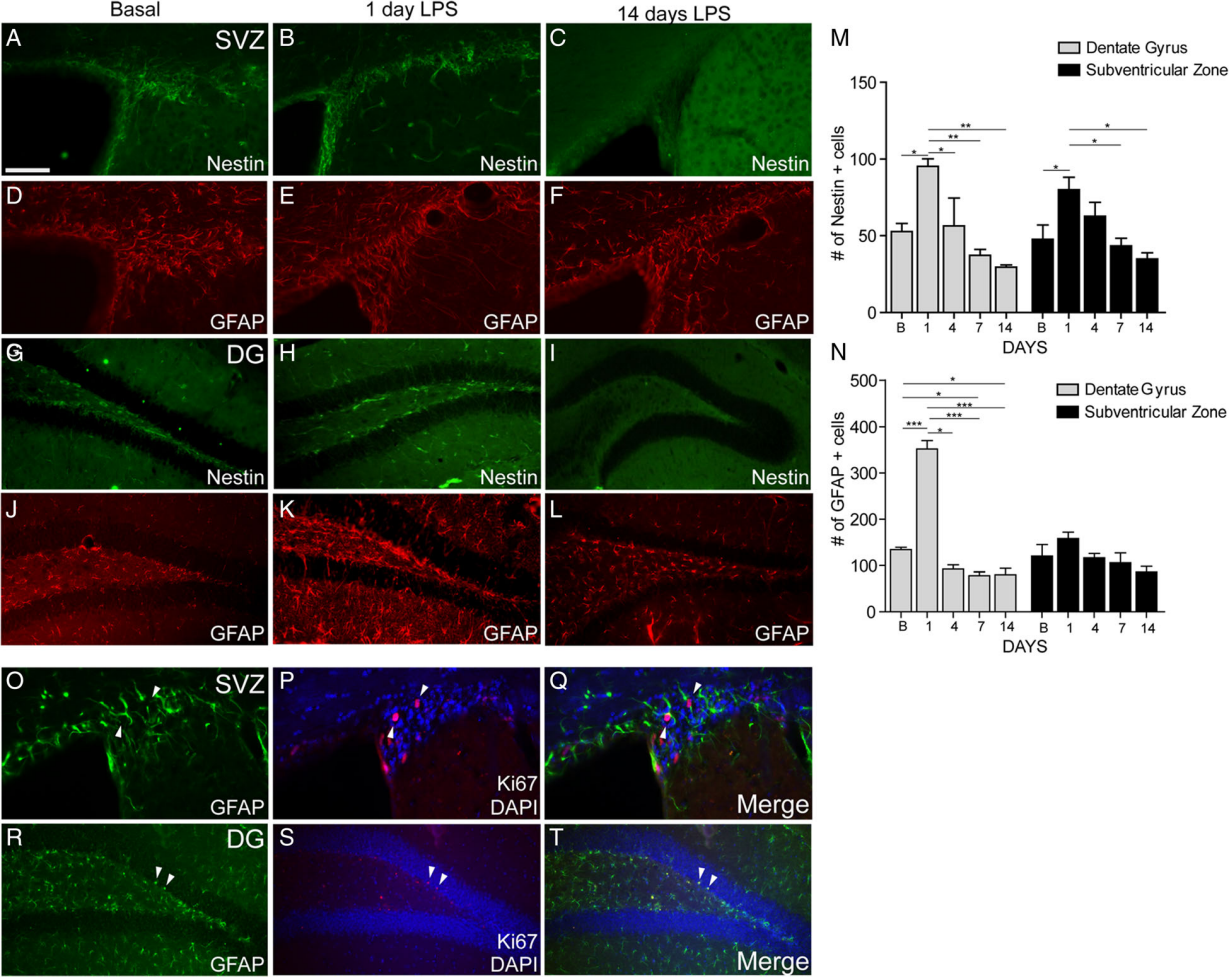
Increased number of GFAP- and nestin-positive cells after acute and chronic LPS administration. Immunofluorescence analysis of nestin and GFAP expression 1 day after LPS showed an increased number of positive cells in the SVZ (**a**, **b**, **d**, **e**) and DG (**g**, **h**, **j**, **k**). 14 days of LPS administration lead to a decrease in the number of NPGs in both neurogenic regions (**c**, **f**, **i**, **l**). All presented panels came from independent staining experiments. Immunofluorescence analysis of GFAP and Ki67 reveals that a small number of Ki67+ cells are also GFAP positives in the SVZ (**m**–**o**) and DG (**p**–**r**) 1 day after LPS. Statistical analysis of nestin- (**s**) and GFAP- (**t**) positive cells have been performed in both the SVZ and DG regions. As by immunofluorescence experiment, a significant increase of nestin and GFAP expression was observed after 24 h after LPS. The *white arrowheads* (**o**–**t**) show the double positive cells. Statistical analysis was performed by one-way ANOVA followed by Tukey’s multiple test *p ≤ 0.05, **p ≤ 0.01, ***p ≤ 0.001 (*n* = 5). *Scale bar*: 100 μm

Contrary to brain response to injuries, including stroke, previous evidence suggests that the acute and chronic inflammation may have a negative impact on neurogenesis [22–27]. To assess the spatial and temporal dynamics of the nestin signal in the context of neuroinflammatory conditions, we used a well-established model of the LPS-induced acute and chronic inflammation. We used two different approaches. The nestin-luc/GFP transgenic mice were injected by single dose of LPS or treated chronically over 14 days (see the “Methods” section) (Fig. 4a–l). The in vivo analysis of bioluminescence revealed that, when compared to baseline levels, a single LPS injection induced a transient but robust increase of the nestin signal peaking 24 h after initial stimuli (Fig. 4a–d). Interestingly, after the single LPS injection, the signal tends to decline after 3 days reaching the baseline levels at 5–7 days (Fig. 4m). In the chronic inflammatory model, the nestin signal was up-regulated after 24 h of LPS and tends to increase after every 3 days (periodical injection) (Fig. 4e–l). However, at the end of the 14 days, the recorded signals were below the baseline levels (Fig. 4m). The cellular expression patterns of nestin were further confirmed by immunofluorescence analysis. The quantitative analysis of the nestin and GFAP expression reveals a marked increase in nestin (Fig. 5a–c, g–i) and GFAP (Fig. 5d, f, j–l) immunoreactivities in the dentate gyrus and in the SVZ 1 day after LPS injection. This increase in immunoreactivities correlates with the increase in the numbers of nestin and GFAP-positive cells in the two regions analyzed. The number of nestin and GFAP-positive cells is significantly increased at day 1 and starts to decrease in the DG and in the SVZ as early as 4 days after LPS injection to reach lower than the basal level at 14 days (Fig. 5m, n). In order to determine whether the observed increase in the number of the GFAP-positive cells is due to an augmentation in proliferation, we performed double immunofluorescence for Ki67 (marker of proliferation) and GFAP 1 day after LPS injection. In fact, very few GFAP cells expressed the proliferation marker Ki67 in the SVZ (Fig. 5o–q) or DG (Fig. 5r–t). Thus, our results suggest that the observed increase in the number of GFAP-positive cells after LPS, at the tested time point, is not due to proliferation [28].

### Inflammation and brain ischemia increase microglial expression of nestin

**Fig. 6:**
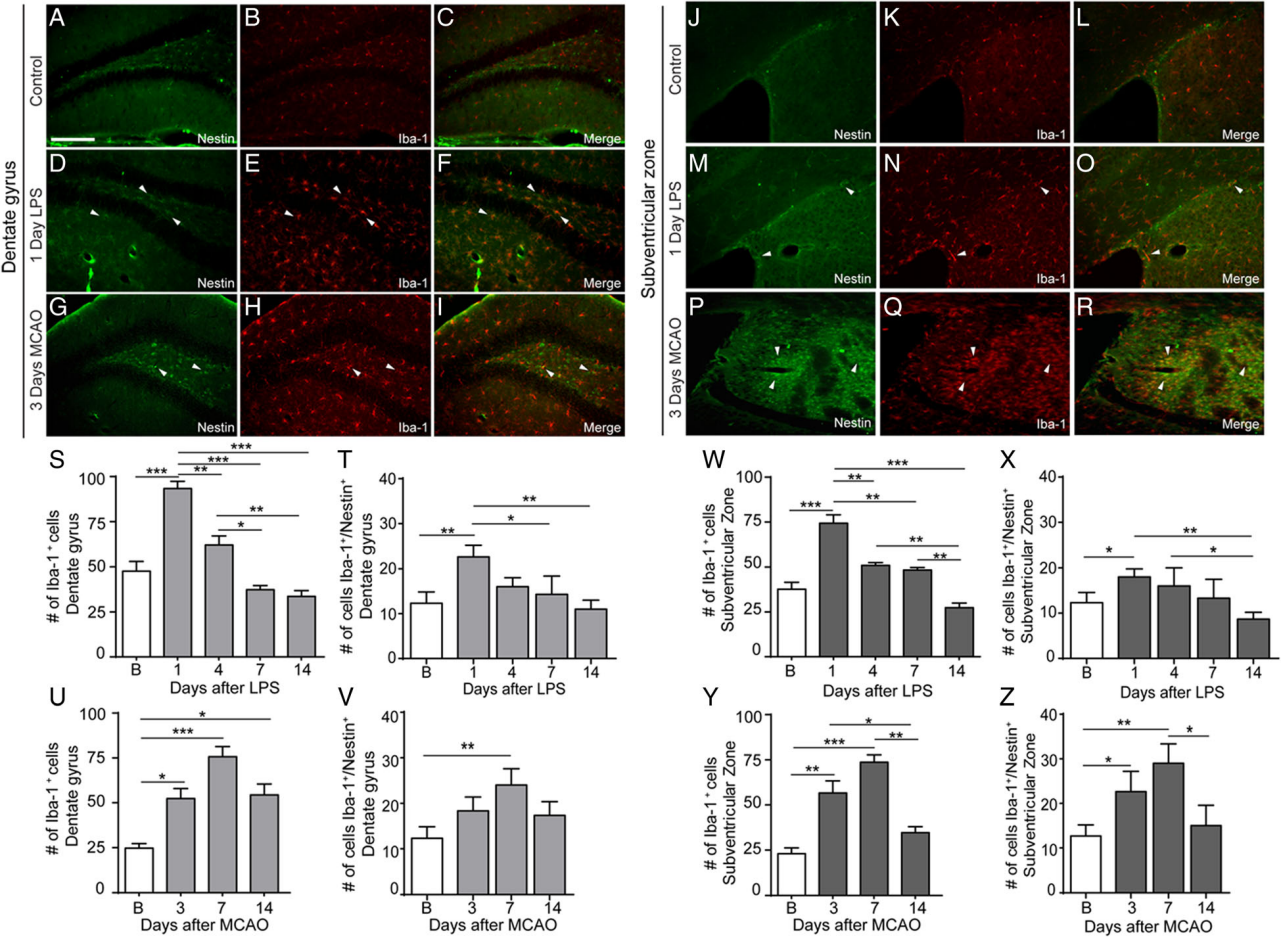
LPS injection showed an increased expression of Iba-1^+^, nestin^+^, and Iba-1^+^/nestin^+^ cells in DG (**a**-**f**) and SVZ (**j**-**o**) when compare to baseline level. Most of the nestin cells are co-localized with Iba-1 positive cells (*white arrowhead*). 3 days after MCAO, Iba-1^+^, nestin^+^ and Iba-1^+^/nestin^+^ cells were observed in DG (**g**-**i**) and in SVZ (**p**-**r**)

While nestin have been widely considered as a putative neuronal stem cell marker, in neuroinflammatory and in post-injury conditions, nestin could also be observed in activated and GFAP-positive astrocytes (see Figs. 3 and 4) [29, 30]. Importantly, the immunofluorescence analysis of the brain sections after stroke revealed several GFP-positive cells with glial morphology that were not positive for the astrocyte marker GFAP. We next asked whether resident glial cells including microglia may express and/or up-regulate nestin in neuroinflammatory conditions. We analyzed activated microglial cells for nestin expression at different time points following LPS challenge and after MCAO. Our areas of interest were the DG and SVZ regions, and since MCAO injury affects the area close to the SVZ, this region also included a peri-infarct zone/stroke area. As expected, the LPS challenge and the inflammatory response following MCAO were characterized by a marked increase in Iba-1 staining (Fig. 6a–r). Interestingly, a double-immunofluorescence analysis revealed that a significant number of activated microglia/macrophages were indeed positive for nestin. As shown in Fig. 6s–z, quantitative analysis of the Iba-1 and the Iba-1/nestin-positive cells revealed that following the LPS challenge, a number of Iba-1/nestin-positive cells showed the highest increase 24 h after the LPS challenge, while following MCAO, the number of nestin-positive microglia/macrophages peaked 7 days after stroke followed by decline at 14 days following initial ischemic injury (Fig. 6u, v, y, z). Note that the SVZ region in the stroke-affected mice also comprises an ischemic area affected by MCAO (Fig. 6p–r). Here it is important to mention that, in the brain, the systemic LPS-induced microglial activation does not lead to infiltration of the peripheral cells; thus, Iba-1 and Iba-1/nestin-positive cells are indeed resident microglia [31]. In the stroke model, however, we cannot exclude that the subset of the nestin/Iba-1-positive cells may be of peripheral origin, thus representing the microglia/macrophage population.

**Fig. 7:**
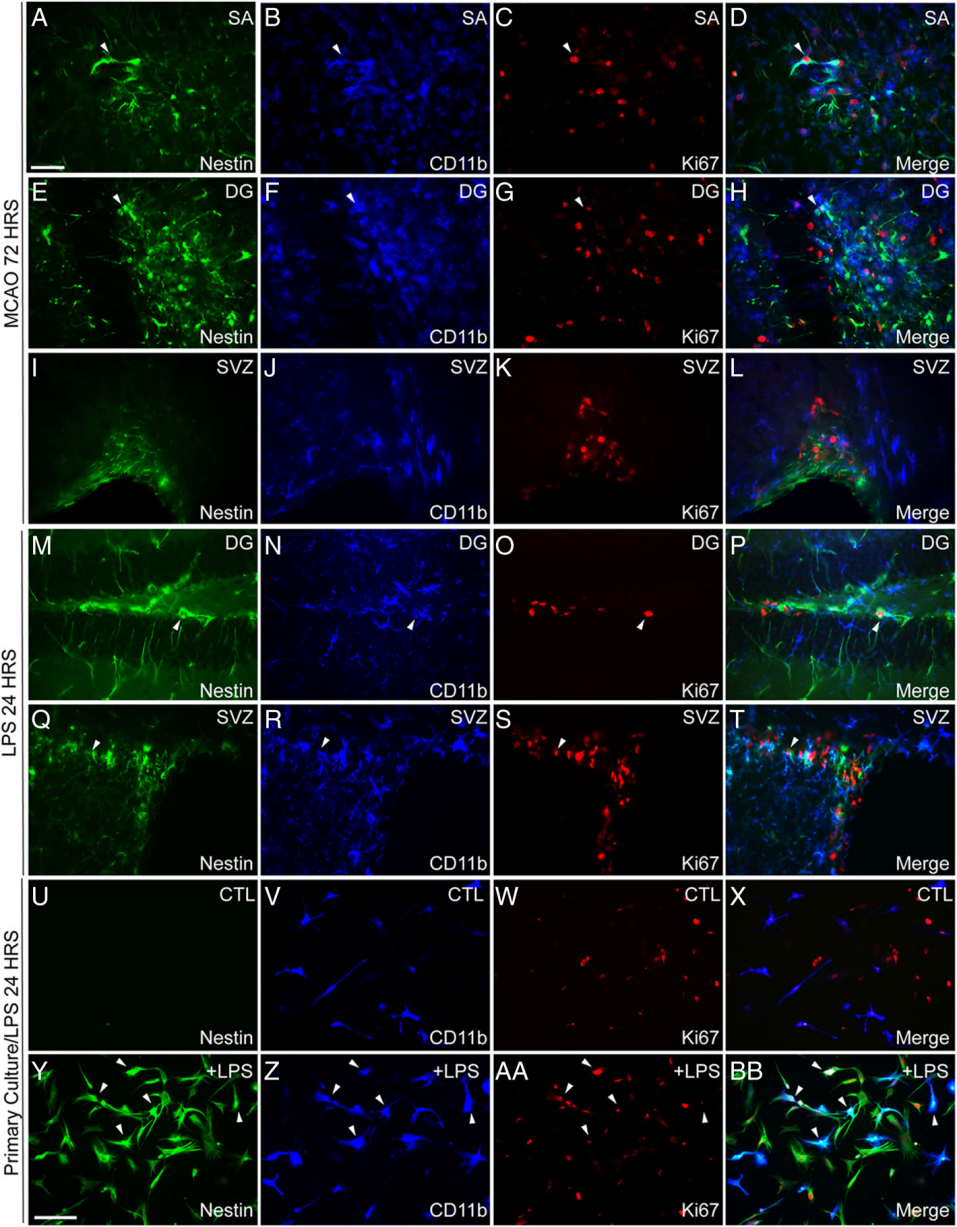
Proliferative response of nestin^+^/Iba-1^+^ cells after inflammatory stimuli. **a**–**l** A strong proliferative response, seen by the immunoreactivity for Ki67, is induced in the brain 72 h after MCAO in the SA (**a**–**d**), DG (**e**–**h**), and SVZ **(i**–**l**) regions. The vast majority of the proliferative cells are CD11b^+^, and very few nestin^+^/CD11b^+^/Ki67^+^ can be found in the 3 regions studied (*white arrowheads*). 24 h after LPS injection, Ki67 immunoreactivity is found in the DG (**m**–**p**) and SVZ (**q**–**t**). The Ki67^+^ cells are either CD11b^+^ or nestin^+^, and very few cells in proliferation express both markers (*white arrowheads*). Primary glial cell culture showed a strong induction of nestin immunoreactivity and proliferation after LPS treatment in CD11b^+^ cells (*white arrowheads*) (**u**–**bb**). *Scale bar*: 50 and 100 μm

One of the important features of microglial activation, especially in the context of ischemic injury, is a robust proliferative response [11, 32]. Thus, we next investigated whether nestin/Iba-1+ cells are indeed dividing microglia. We performed immunofluorescence analysis for Ki67 (marker of proliferation), nestin, and Iba-1. Given the temporal differences in the peak numbers of the Iba-1-positive cells following LPS challenge and MCAO (see Fig. 6), identification analysis of the Iba-1/nestin/Ki67-positive cells was performed either 24 h (LPS) or 72 h (MCAO) following initial brain challenge. As revealed in Fig. 7, following stroke, only few Iba-1/nestin/Ki67-positive cells were detected in the stroke area (SA), DG, and SVZ 72 h after MCAO (Fig. 7a–p). The similar findings were obtained following LPS challenge (Fig.7m–t). Namely, we identified only few Iba-1/nestin/Ki67-positive cells in the DG and SVZ. To further confirm that microglial cells up-regulate nestin in inflammatory conditions, we performed additional series of in vitro experiments using primary glial cultures, enriched in microglia. Indeed as revealed in Fig. 7u–bb, the LPS treatment was associated with a robust induction of nestin in CD11b-positive cells. In addition, the triple Iba-1/nestin/Ki67 staining revealed that some of the nestin-positive microglial cells were dividing (Fig. 7bb).

Taken together, our results strongly suggest that following injuries and in the context of acute innate immune challenge, in addition to reactive astrocytes, activated Iba-1 microglia/macrophages strongly up-regulate nestin. This suggests that nestin may serve as a context-dependent marker of activated microglia/macrophages.

## Discussion

Nestin, a class VI intermediate filament, is a well-established putative neuronal stem cell marker. Here we describe the nestin-luc/GFP reporter mouse model as a novel transgenic tool for real-time analysis and/or visualization of the nestin signals from the brains of living animals. Using in vivo biophotonic/bioluminescence imaging, we were able to visualize the baseline levels, as well as the spatial and temporal dynamics of the nestin biophotonic signal induction following ischemic injury and in the experimental paradigms of the acute and chronic neuroinflammation. Here it is important to mention that in all experimental paradigms, analysis of the fluorescence GFP transgene signal at the cellular level revealed a co-expression between endogenous nestin and the nestin-driven GFP transgene. This suggests that nestin-luc/GFP reporter mouse represents a valid model system for real-time analysis of nestin induction and/or expression patterns. By combining in vivo bioluminescence/biophotonic imaging with double immunofluorescence, we report here that in baseline, physiological conditions, in the adult brain, nestin expression is indeed restricted to neuronal progenitor cells. Thus, the obtained in vivo biophotonic signal can be used to assess neurogenic activity from the brains of adult mice. Importantly, ischemic brain injury as well as controlled innate immune challenge induced significant increase of the nestin signal in vivo. Contrary to physiological conditions where the GFP signal co-localized perfectly with nestin-positive progenitors, the double-immunofluorescence analysis of the nestin-driven transgene gfp revealed, however, that in neuroinflammatory conditions, the cellular expression and/or induction of the nestin biophotonic signal was not restricted to neuronal progenitors. As expected, we observed a marked increase in nestin expression/induction in the GFAP-positive reactive astrocytes. However, to our surprise, following LPS challenge as well as after MCAO, we observed a significant increase in the nestin expression in activated Iba-1 microglia/macrophages (see Figs. 5 and 6).

Brain injuries and neurodegenerative disorders are associated with acute and chronic brain inflammation. The relationship between brain inflammation and the regulation of neurogenesis has been well documented and remains the subject of intense investigation. Notably, recent collective evidence indicates that neurogenesis is affected during brain injury and in distinct neuroinflammatory conditions by the dysregulation of cytokines, chemokines, neurotransmitters, and reactive oxygen species caused by inflammation and mediated by activated macrophages, microglia, and reactive astrocytes [33–42]. Multiple models of ischemic-induced inflammation have demonstrated an increase in neurogenesis [33–42]. On the other hand and contrary to ischemic injury that has been associated with a strong induction of neurogenesis in the SVZ and DG area, inflammation as well as irradiation have been generally thought to disrupt progenitor cell proliferation and adult neurogenesis [43]. In our previous work, we and others have shown that doublecortin-positive cells in baseline conditions as well as after ischemic injury express innate immune receptors suggesting an intense dialog between the immune system and NPGs [6, 7]. Nestin is a putative marker of NPGs; importantly, however, nestin expressing NPGs in addition to new neurons may give a rise to a wide variety of cells including astroglia and oligodendrocytes [2, 3, 44]. Thus, we hypothesize here that neuroinflammatory conditions and injuries may significantly affect and/or contribute to a differentiation of the nestin-positive cells and thus induce the shift towards gliosis.

A novel transgenic tool that we generated in our laboratory allowed us to investigate the role and/or spatial and temporal dynamics of the nestin expressing cells in brain response to injury and in neuroinflammatory conditions. For example, a great advantage of our in vivo model system is that it allowed us to track how nestin expressing cells are responding to different types of neuroinflammatory conditions directly from the brains of intact animals (see Figs. 3, 4, and 5). On the other hand, a co-expression of the fluorescence reporter GFP permitted identification of the cells expressing nestin following different stimuli. As expected, and in accordance with the previous work [45], MCAO was associated with a marked increase in nestin expressing reactive astrocyte. The similar induction pattern was observed following LPS stimuli however with slightly different temporal dynamics (see Figs. 4 and 5). Importantly, a chronic exposure to LPS resulted in marked decrease in the nestin biophotonic/bioluminescence signal as well loss of immunostaining. Interestingly, during our analysis, we observed a number of cells having glial morphology expressing nestin-driven transgene GFP but were not GFAP positive. In keeping with recent and rather intriguing findings by Elmore and colleagues [4] describing that in microglia-depleted brain, repopulation of microglia occurs through proliferation of nestin-positive cells, we next asked whether the unidentified nestin expressing cells are indeed activated microglia/macrophages. To our surprise, the analysis of immunostaining, in both neuroinflammatory conditions, revealed a subpopulation of the Iba-1/nestin-positive cells. Although the origin of the nestin expressing microglia remains unclear [46], the increase in Iba-1/nestin-positive cells was more pronounced after stroke, where 7 days after injury, approx. 40% of activated microglial cells were nestin positive (Fig. 6). Hence, our data suggest that in addition to reactive astrocytes in response to injury and neuroinflammation, activated microglial cells may express stem cell markers like nestin.

## Conclusions

In summary, our results suggest that neuroinflammatory conditions such as brain ischemia and innate immune challenge strongly up-regulate nestin signals in the brain of living animals. Interestingly, while in physiological conditions, and in the adult brain, nestin expression is restricted to NPGs in their respective niches and the neuroinflammatory conditions are associated with marked induction of nestin in astrocytes as well as in activated microglia/macrophages. Based on our results, we propose that nestin may serve as a context-dependent biomarker of inflammatory response in glial cells including activated brain microglia/macrophages.

## Additional file

Additional file 1: Figure S1. Negative controls. Representative photomicrographs of imaging of WT mice in baseline (A), 24 h after MCAO (B), and 24 h after LPS (C). No bioluminescence signal can be observed in the three groups of experimental animals confirming the specificity of the signal emission in the nestin-luc-GFP mice. No GFP immunoreactivity can be observed in the brain sections of WT mice and 24 h after stroke and LPS (D–I), and no unspecific staining can be observed with the secondary antibody, Alexa 594 anti-mouse, used in this study (J, K). Note that DAPI staining is added to visualize neuroanatomical region (DG) of the negative controls. Scale bar: 100 μm. (TIF 2419 kb)

## Abbreviations

CNS: Central nervous system
DG: Dentate gyrus
DLIT: Diffuse light imaging tomography
GFAP: Glial fibrillary acidic protein
GFP: Green fluorescent protein
LPS: Lipopolysaccharide
Luc: Luciferase
MCAO: Middle cerebral artery occlusion
NPGs: Neuronal progenitors
TLR2: Toll-like receptor 2
WT: Wild type
