# Supplementary figures and images for: Molecular imaging of nestin in neuroinflammatory conditions reveals marked signal induction in activated microglia
Source: J Neuroinflammation. 2017 Mar 3;14:45. doi: 10.1186/s12974-017-0816-7 (PMC5335711; doi:10.1186/s12974-017-0816-7)

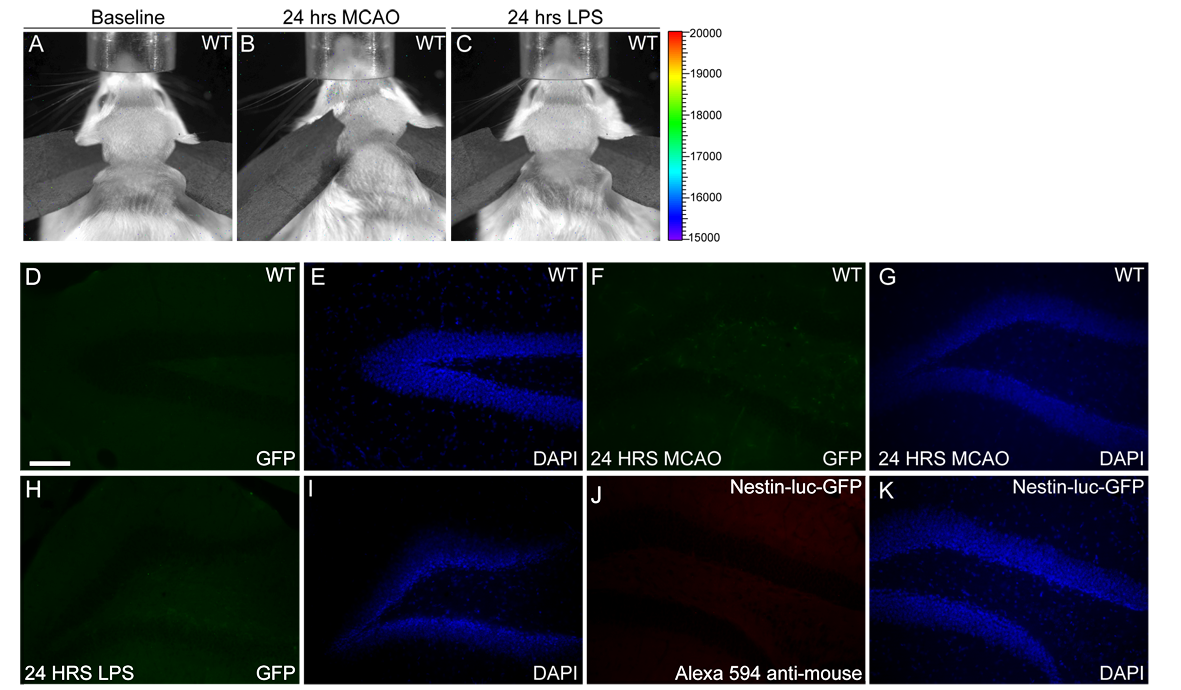

Supplement: Additional file 1: Figure S1. — Negative controls. Representative photomicrographs of imaging of WT mice in baseline (A), 24 h after MCAO (B), and 24 h after LPS (C). No bioluminescence signal can be observed in the three groups of experimental animals confirming the specificity of the signal emission in the nestin-luc-GFP mice. No GFP immunoreactivity can be observed in the brain sections of WT mice and 24 h after stroke and LPS (D–I), and no unspecific staining can be observed with the secondary antibody, Alexa 594 anti-mouse, used in this study (J, K). Note that DAPI staining is added to visualize neuroanatomical region (DG) of the negative controls. Scale bar: 100 μm. (TIF 2419 kb) [file 12974_2017_816_MOESM1_ESM.tif]
